# Role of cytokine in malignant T-cell metabolism and subsequent alternation in T-cell tumor microenvironment

**DOI:** 10.3389/fonc.2023.1235711

**Published:** 2023-09-07

**Authors:** Megha Yadav, Blessi N. Uikey, Shantnu Singh Rathore, Priyanka Gupta, Diksha Kashyap, Chanchal Kumar, Dhananjay Shukla, Vijayamahantesh, Arvind Singh Chandel, Bharti Ahirwar, Ashish Kumar Singh, Shashi Shekhar Suman, Amit Priyadarshi, Ajay Amit

**Affiliations:** ^1^ Department of Forensic Science, Guru Ghasidas Vishwavidyalaya, Bilaspur, India; ^2^ Department of Biotechnology, Guru Ghasidas Vishwavidyalaya, Bilaspur, India; ^3^ Department of Immunology and Microbiology, University of Missouri, Columbia, SC, United States; ^4^ Center for Disease Biology and Integrative Medicine, Faculty of Medicine, The University of Tokyo, Bunkyo, Japan; ^5^ Department of Pharmacy, Guru Ghasidas Vishwavidyalaya, Bilaspur, India; ^6^ Department of Microbiology, RK University, Rajkot, India; ^7^ Department of Zoology, Udayana Charya (UR) College, Lalit Narayan Mithila University, Darbhanga, India; ^8^ Department of Zoology, Veer Kunwar Singh University, Arrah, India

**Keywords:** T cell lymphoma, metabolism, cytokine, tumor micro environment, cell signaling

## Abstract

T cells are an important component of adaptive immunity and T-cell-derived lymphomas are very complex due to many functional sub-types and functional elasticity of T-cells. As with other tumors, tissues specific factors are crucial in the development of T-cell lymphomas. In addition to neoplastic cells, T- cell lymphomas consist of a tumor micro-environment composed of normal cells and stroma. Numerous studies established the qualitative and quantitative differences between the tumor microenvironment and normal cell surroundings. Interaction between the various component of the tumor microenvironment is crucial since tumor cells can change the microenvironment and vice versa. In normal T-cell development, T-cells must respond to various stimulants deferentially and during these courses of adaptation. T-cells undergo various metabolic alterations. From the stage of quiescence to attention of fully active form T-cells undergoes various stage in terms of metabolic activity. Predominantly quiescent T-cells have ATP-generating metabolism while during the proliferative stage, their metabolism tilted towards the growth-promoting pathways. In addition to this, a functionally different subset of T-cells requires to activate the different metabolic pathways, and consequently, this regulation of the metabolic pathway control activation and function of T-cells. So, it is obvious that dynamic, and well-regulated metabolic pathways are important for the normal functioning of T-cells and their interaction with the microenvironment. There are various cell signaling mechanisms of metabolism are involved in this regulation and more and more studies have suggested the involvement of additional signaling in the development of the overall metabolic phenotype of T cells. These important signaling mediators include cytokines and hormones. The impact and role of these mediators especially the cytokines on the interplay between T-cell metabolism and the interaction of T-cells with their micro-environments in the context of T-cells lymphomas are discussed in this review article.

## 1 Introduction

T-cell lymphoma (TCL) is a subclass of non-Hodgkin lymphoma (NHL) (1). The diagnosis and identification of risk factors associated with TCL are challenging due to the lower number of cases. TCLs cover a wide range of conditions that differ in disease progression and clinical symptoms. Broadly, TCLs are classified into peripheral T-cell lymphoma (PTCL) and cutaneous T-cell lymphoma (CTCL) (1) (**Table 1**). TCLs account for only one-sixth of all NHLs, and most of them are of PTCL type (4). The basis of differentiation between PTCL and CTCL may be phenotypic, diagnostic, or prognostic (5). Over the years, this classification has been updated by the Revised European American Lymphoma (REAL) classification and the European Organization of Research and Treatment of Cancer (EORTC) (6). REAL is a WHO project which recently updated the categorization of tumors of lymphoid and hematopoietic origin (7). This updated classification identifies around 30 different types of PTCLs (7, 8). CTCL lymphomas are present on the skin with an absence of any subcutaneous symptoms at the time of diagnosis (6, 9). Clinically, CTCLs may have a slow or very aggressive mode of prognosis (6, 9, 10). Treatment and diagnosis of TCLs require a multidisciplinary approach inclusive of short-term or long treatment goals (9, 10).

**Table 1:** WHO classification of T-cell lymphoma (source reference: 2, 3).

Types of T-cell lymphomas	Phenotypic expression	TCR type
Leukemic		
T-cell prolymphocytic leukemia	Non-cytotoxic	Tαβ
T-cell large granular lymphocytic leukemia	Cytotoxic (Expression of perforin and/or granzyme B in addition to TIA-1)	Tαβ (more rarely Tγδ)
Chronic lymphoproliferative disorders of NK cells*	Cytotoxic (Expression of perforin and/or granzyme B in addition to TIA-1)	NK
Aggressive NK-cell leukemia	Cytotoxic (Expression of perforin and/or granzyme B in addition to TIA-1)	NK
Systemic EBV-positive T-cell lymphoproliferative disease of childhood	Cytotoxic (Expression of perforin and/or granzyme B in addition to TIA-1)	Tαβ
Adult T-cell leukemia/lymphoma	T-regulatory	Tαβ
Nodal		
Peripheral T-cell lymphoma, not otherwise specified	Variable, a subset T_FH_, a subset Cytotoxic (Expression of perforin and/or granzyme B in addition to TIA-1)	Tαβ (CD4>CD8), rarely Tγδ
Angioimmunoblastic T-cell lymphoma	Tfh	Tαβ (CD4, T_FH_)
Anaplastic large-cell lymphoma, ALK-positive	Cytotoxic (Expression of perforin and/or granzyme B in addition to TIA-1)	Tαβ (Likely Th_2)_
Anaplastic large-cell lymphoma, ALK-negative	Cytotoxic (Expression of perforin and/or granzyme B in addition to TIA-1)	Tαβ
Cutaneous		
Mycosis fungoides	Non-cytotoxic	Tαβ (mostly CD4)
Sézary syndrome	Non-cytotoxic	Tαβ (mostly CD4)
Primary cutaneous CD30+ T-cell lymphoproliferative disorders 1. Primary cutaneous anaplastic large cell lymphoma 2. Lymphomatoid papulosis	Cytotoxic (Expression of perforin and/or granzyme B in addition to TIA-1) Cytotoxic (Expression of perforin and/or granzyme B in addition to TIA-1)	Tαβ (mostly CD4) Tαβ (CD4) Tαβ (CD4)
Subcutaneous panniculitis-like T-cell lymphoma	Cytotoxic (Expression of perforin and/or granzyme B in addition to TIA-1)	Tαβ (CD8)
Primary cutaneous γδ T-cell lymphoma	Cytotoxic (Expression of perforin and/or granzyme B in addition to TIA-1)	Tγδ (Vδ2)
Primary cutaneous CD8+ aggressive epidermotropic cytotoxic T-cell lymphoma*	Cytotoxic (Expression of perforin and/or granzyme B in addition to TIA-1)	Tαβ (CD8)
Primary cutaneous CD4+ small/medium T-cell lymphoma*	T_FH_	Tαβ (CD4, T_FH_)
Hydroa vacciniforme-like lymphoma	Cytotoxic (Expression of perforin and/or granzyme B in addition to TIA-1)	Tαβ (rarely NK)
Extranodal		
Extranodal NK/T-cell lymphoma, nasal type (ENKTCL)	Cytotoxic (Expression of perforin and/or granzyme B in addition to TIA-1)	NK (more rarely Tγδ or Tαβ)
Hepatosplenic T-cell lymphoma (HSTL)	Cytotoxic—expression of TIA-1 only	Tγδ (Vδ1) (more rarely Tαβ
Enteropathy-associated T-cell lymphoma (EATL)	Cytotoxic (Expression of perforin and/or granzyme B in addition to TIA-1)	IEL, Tαβ (more rarely Tγδ)

With the recent advancement in the field of metabolomics or lipidomics, the understanding of cancer metabolism has increased. Developing tumors requires high energy and the macromolecule building block to support not only their proliferation but also the adaptation to changing environments during the process of metastasis (11–13). The Warburg effect along with increased glucose uptake is the characteristic metabolic feature of tumor cells (11–13). To fulfill these requirements, the cancer cell metabolism must be reprogrammed (13, 14). The activated T-cell metabolism resembles cancer cell metabolism up on activation through T-cell receptor signaling accompanied by co-stimulation through CD28 induction (15). To sustain this active state, IL-2-mediated signaling is a must (16). Similar to tumor cells, post-activation T cells divide rapidly much faster than any somatic cell (15, 17). Metabolically active T cells reprogram their metabolism to increase the uptake and utilization of glucose accompanied by changes in calcium flux with upregulated lactate production (18, 19). T-cell receptor (TCR)-mediated signaling starts glucose transport upregulation, but to attain the maximum metabolic reprogramming, CD28-mediated signaling must be activated (19, 20). In addition to TCR and costimulatory signaling, cytokine-mediated signaling is of utmost importance for the development of effector T cells, which resembles T-lymphoma cells metabolically (15, 21).

Irrespective of these metabolic similarities, the activated T cells rarely become cancerous, and once the immune challenges are over, the activated T cell either goes to apoptosis or attains the quiescent memory T-cell state (22–24). In the case of TCLs or other lymphomas, instead of going to apoptosis or resting memory cell state, the T lymphocyte remains in the state of hyperactivity due to gain-of-function mutation in the oncogene or loss of function of the tumor-suppressor gene (22, 25). This continuous state of proliferation is associated with altered metabolism in comparison with normal T cells (19, 25). Various reports associated the different genetic alternations in genes related to cytokine signaling with the pathogenesis of TCLs (25–30). Interactions between lymphoma cells and their microenvironment mediated by the altered cytokine profile of the tumor microenvironment (TME) disturb tissue homeostasis and thus favor lymphoma development and progression (31–34). The various aspects of T-cell lymphoma development like metastasis, migration, invasion apoptosis, and epithelial–mesenchymal transition (EMT) are regulated by the altered metabolic phenotype of T lymphoma cells and their abnormal interaction with other non-neoplastic cellular components of the TME (22, 24, 31). As with other solid and hematologic cancers, TCL progenesis depends upon the nature of tumor-infiltrating lymphocytes (TiLs). These TiLs are different from the original lymphoid organ immune cell and are predominantly T lymphocytes. It has been postulated that the interaction between TiLs and T lymphoma cells dictates the subsequent disease progression (35, 36). The TME is ever changing due to the entry of TiLs and subsequent inflammation associated with it (35–37). It has been reported that these TiLs induce inflammation and help in tumor cell survival (35).

For the proper functioning of the immune system, intercellular communication between immune cells is crucial. The important mediator of this immune communication is cytokine, a protein with low molecular weight. Cytokines are mainly secreted by immune cells and stromal cells and have a regulatory role in immune cell activation and subsequent differentiation, proliferation, and apoptosis (38). The role of cytokine in TCL progression is not very clear and depends upon the TME. The effect of cytokine may be either antitumor or malignancy promoting, which depends on the ratio of pro- and anti-inflammatory cytokines along with the level of expression of cytokine receptors present in the TME (39, 40). Since cytokines are a very important cog in the wheel of cellular communication, this review is aiming to discuss their role in the metabolism of T-cell lymphomas and the subsequent development of the tumor microenvironment.

## 2 Tumor microenvironment of T-cell lymphoma and the role of cytokine and other signaling pathways in cross-talk between its cellular constituents

**Figure 1:**
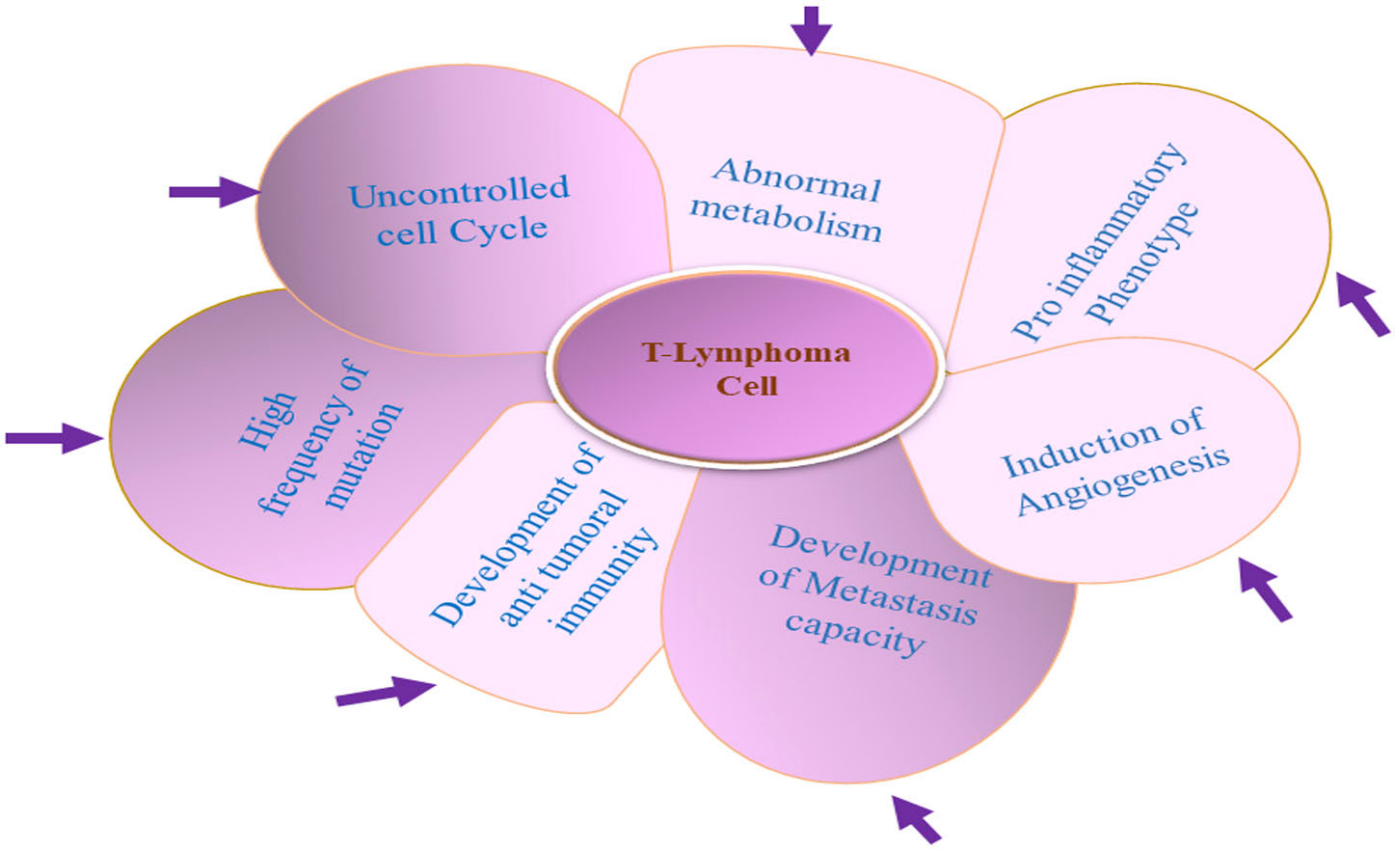
Unusual properties of T-lymphoma cell: T-lymphoma cell’s unusual properties is dictated by an interwoven complex signaling network between T lymphoma cell and other normal constituent cells of the tumor microenvironment (TME).

The tumor cell microenvironment (TME) is a heterogenous body constituted of tumor cells, normal resident cells, infiltrating immune cells, extracellular matrix, and a variety of secreted mediators (31–34, 41, 42). The normal cells of the TME help in tumor promotion, and the intercellular communication inside the TME is mediated through the interwoven complex network involving various cytokines and enzymes, along with a plethora of growth factors (43–47). To understand the cancer disease mechanism, elucidation of this intercellular signaling is of utmost importance. T-cell lymphomas are generally highly proliferative and resistant to apoptosis and accumulate in the nodal and particular extranodal sites (48–51). Despite the general resistance to chemotherapy, culturing T-cell lymphoma cell lines for long is difficult because these cell lines are very prone to spontaneous apoptosis in *in vitro* conditions (52). These observations established the role of the TME in the pathogenesis of T-cell lymphomas (35–37). The TME is the main source of extrinsic growth, and the survival signal provided by the TME’s non-neoplastic cells including different hematopoietic (myeloid, lymphoid) and non-hematopoietic cells is essential for T-lymphoma survival (35–37) (**Figure 1**). The source T-cell variant from which the tumorigenic T cell derives significantly dictates the composition of the TME (39, 42, 53, 54). As in the case of AITL (angio-immunoblastic T-cell lymphoma), the overexpression of follicular T helper cells (Tfh) associated with cell surface receptors (ICOS, PD-1, and CXCR-5) along with BCL-6 established that AITL tumorigenic cells originated from Tfh cells. The cytokines (CXCL13, LT-beta, and IL-21) influence the recruitment, functional polarization, and expansion of cells within the TME of AITL (49, 55–57). While B-cell recruitment in the TME is promoted by CXCL-13 and IL-21, recruitment of follicular dendrite cells has been promoted by LT-β (58–60). The unique expression portfolio of different subsets of T-lymphoma cells for the cell surface ligand, cytokines, and chemokines provides uniqueness to its respective TME (25, 37, 61). The uniqueness of the TME of different subtypes of TCL is regulated by unique transcription factors specific for each subset of T cells (36, 61–63).

Recently, it has been found that in non-Tfh-derived T-cell lymphomas like PTCL-NOS, gene regulators like t-bet, GATA-3, and FoxP3 regulate the differential expression of their target genes including those cytokines that regulate the constituents of the TME (64–66). These observations suggested that the cell of origin is a major factor behind the composition of the TME in developing T-cell lymphomas and established the importance of TCR-mediated signaling aided by co-stimulatory and cytokine signaling in transcriptional and posttranscriptional regulations of these master transcription factors (38). These signaling alternations subsequently result in the development of a specific TME, which is evident by the homology between the receptor profile of T-lymphoma cells and the T cells’ source of origin subtypes (53, 54). Subsequently, the constituents of the TME in turn influence the availability of ligands and cytokines which drive the T-cell lymphoma growth proliferation and survival.

### 2.1 Cytokine and cell signaling in the tumor microenvironment of cutaneous T-cell lymphomas

As discussed in the above section, the nature and composition of the TME depends upon various inter- and intracellular signaling pathways. The TME associated with hematologic tumors vastly differs from the solid tumor microenvironment (67). Mycosis fungoides (MF) is the most frequent type of CTCL, whereas the leukemic variant of CTCL, Sezary syndrome (SS), is the rarest (67). Apart from T-cell transformation progression, MF depends on the interactions between T cells with other cellular components of its TME like antigen-presenting cells (68). It has been established that the interaction between T lymphomas and various components of the microenvironment is regulated by specific cell signaling, and to understand the T-cell abnormalities in CTCL (38), deciphering the mechanism of these signaling pathways is crucial. Alterations in these signaling networks promote malignant T-cell proliferation, survival, T-cell migration, inflammation, and suppression of immune regulation of malignant T cells, resulting in the development of a skin microenvironment that allows disease progression (69, 70). Targeting key proteins like ITK, PI3K, SYK, and mTOR of signaling pathways which are under clinical trials in other disease conditions may open the way for the development of new therapeutic options for CTCL treatment (38). Analyzing the signals responsible for the interaction of T-lymphoma cells with other cells and the extracellular matrix of the microenvironment is important for the identification of new molecular therapeutics molecules (31, 35).

With the help of recently developed high-throughput technologies, various important genetic modulations in cytokine signaling-associated genes have been identified in MF (71–73). The overexpression of downstream signaling components like MAPK of cytokine receptors is due to gain-of-function mutation in B-Raf (p. Asp594Asn) and ERK-1 (p. Glu322Ala, p. Glu322lys) in MF subjects (74). In stage IV subjects of MF, mutations were identified in KRAS (Kristen Rat Sarcoma Viral oncogene homolog) and NRAS (Neuroblastoma RAS Viral oncogene homolog) genes (74–76). Immunohistochemistry studies revealed that in the nucleus of 53% cells of MF lesion, ERK1/2 gets phosphorylated, which has been associated with phospho-4E-BP1 (Eukaryotic Translation Initiation Factor 4E Binding Protein 1) (p-4E-BP1) upregulation. Phospho-4E-BP1 (p-4E-BP1) is an important component of PI3K/Akt downstream signaling (69, 77, 78), and the PI3/Akt pathway is important to support metabolic alternation in the TME. Phosphorylation of Akt, an important component associated with cytokine downstream signaling, is a clear indicator of poor patient survival, and the level of phosphorylated PTEN (Phosphate and TENsin homolog) (inhibitor of PI3K-downstream signaling) is inversely associated with the later clinical phase of MF (69, 78–80). Alternation in PI3K-related genes involved in TCR-CD28 signaling along with deletion in PTEN was found through whole-exome sequencing of MF and SS subjects in comparison with healthy skin tissue (81, 82). Mutation in PTEN regulatory proteins such as p. Arg297Cys in PREX2 has also been reported in MF (69, 83). It has also been reported that upregulation of 14 miR-122 occurred in advanced-stage MF, which has been correlated with a decrease in chemotherapy response via Akt activation along with p53.1 inhibition (84).

It has been reported that mutation altering the gene of MAPK and the PI3K/Akt/mTOR axis is crucial for cell metabolism and rarely results in alternation in the amino acid sequence of the protein (85, 86). Therefore, the reason behind the upregulation in these signaling pathways may be attributed to changes in the TME of CTCL. Activation of T-cell lymphoma may produce ligands that can activate this signaling pathway. It has been reported that IL-2 levels in the TME of CTCL get upregulated, which is inducive for the ERK1/2 pathway (87, 88). The exosomes containing proteins of signaling pathway RAS-MEK secreted in response to T-cell activation may further induce ERK-1/2 activation in a paracrine way (47, 69, 89). In addition to these, triggering other signaling pathways downstream of cytokine receptors like NF-κ β and JAK/STAT can result in the activation of MAPK and PI3K/Akt signaling in the TME of CTCL (69, 90–92). It has been established that these pathways are crucial for metabolic alternation to support TCL progression. The ERK-1/2 and PI3K/Akt/mTOR pathways downstream of cytokine signaling cross-talk among themselves and may work together synergistically in the TME of CTCL (38, 69, 92–95). It has been reported that simultaneous induction of nuclear p-ERK, P-Akt, and p-mTOR takes place in 27% of MF lesions (69). Various genetical studies reported that there are changes mainly in the T-cell receptor (TCR) and tumor necrosis factor receptor (TNFR) which have been correlated with the survival and proliferation of tumorous T cells (38). Genetic alternation in these receptors invariably affects TCR-mediated signaling, TCR complex assembly formation, and the level of various proteins such as TCR-associated enzymes, along with various transcription factors. All these are important for the proper execution of downstream signaling mediated through these receptors (8, 26, 26, 38, 50, 76, 83, 96, 97). It has been found that enhanced TCR activation through gain-of-function mutation in the extracellular domain of CD28 and the translocation of CTLA4-CD28 occurred in CTCL (98, 99).

In general, tumor cells bring immune suppression in the TME through the programmed cell death protein 1 (PD-1)-mediated signaling pathway induced by its ligands, programmed death ligands 1 and 2 (PD-L1 and PD-L2) (100). Expression of PD-1 occurs predominantly on activated immune cells like T cells, dendritic cells, natural killer cells, and B cells as well as various types of tumor cells including TCL-expressed PD-1 ligand PD-L1 (82, 101–103). The anti-PD-L1 antibody has shown antitumor response in cancer patients with high levels of PD-L1 expression on tumor-infiltrating immune cells, and with some difference in results, a high expression of PD-1 has been observed in CTCL too (82, 104, 105). This high level of expression of PD-1 is associated with CTCL progression as the level of expression of PD-1 and PD-L1 is higher in an advanced stage in comparison with better-managed CTCL (82, 103). It has been reported that PD-1/PD-L1 inhibition through specific antibodies downregulates tumor progression in mice with enhanced IFN-γ production. The same study reported the objective response rate (ORR) of 15 and 38% in MF when treated with nivolumab in MF and pembrolizumab, respectively (106, 107). It has been reported that transcription factor NF-κ β , which is important for TCR-mediated immune response, gets activated in CTCL (108, 109). Various kinds of mutations such as somatic, splice site, heterozygous deletions, and truncations have been reported in genes like NF-κ β 2 and CARD11, which are crucial for NF-κ β signaling (98, 108, 109). Another possible mechanism for activation of the NF-κ β pathway is through tumor necrosis factor receptor 2 (TNFR2, encoded by TNFRSF1B) (38, 110, 111). The ligand for TNFR2 is TNF-α, and in the case of CTCL, point mutation in TNFRSF1B was observed along with amplification of the related gene (38, 111). An inhibitor of TNF-α-induced NF-κ β is TNF-α-induced protein 3 (TNFAIP3), which has been downregulated in some SS subjects (69, 112). Kinases like TAK1 are the main regulator of NF-κ β , and the level of TAK1 has been reported to be upregulated in CTCL, but inhibition of TAK1 hampers the NF-κ β pathway, resulting in the induction of apoptosis in the CTCL cell line in both *in vitro* and *in vivo* conditions (69, 113, 114). Toll-like receptors (TLRs) are a class of pattern-recognition receptors comprising more than 10 surface receptors important for pathogen recognition. The signaling mediated through TLRs and subsequent induction of cytokine release are important in various skin pathologies including MF (115, 116). Treatment with TLR9 stimulators such as cytosine-phosphate-guanine (CpG) and oligodeoxynucleotides induces Th1-inducing cytokines, which have been proven to be clinically significant in MF and SS subjects (115, 117, 118). In addition to that, the agonist of TLR7/8 is very useful in clearing MF-associated psoralen and ultraviolet A-resistant plaques (119, 120). In an independent trial with patients of MF, imiquimod, an immunomodulatory agent, had given positive results (121).

UV-induced transition plays an important role in the development of MF, and transitions have been represented by both C>T and CC>>TT mutations (122–124). There are three specific mutations associated with UV exposure that have been reported in MF, and furthermore, the most frequent (41%) exotic mutations are C to T transitions in MF (125–127). This finding indicates a link between exposure to UV light and genetic changes in the MF microenvironment. These exposures seem to be a gain-of-function mutation in proto-oncogenes along with loss of function in tumor suppressors and an unusual expression of cytokines and adhesion proteins (122–127). The C>T transition in the P53 gene is a UV signature mutation (128) that is very crucial for tumor progression and is the most frequent gene mutation associated with CTCL, but this mutation is more frequent in the later stage of cancer than the early tumor stage in MF (125, 126). Mutations in important cell-cycle regulators like RB1 and CDKN1B/CDKN2A are also common in CTCL (69, 129). A sufficient supply of oxygen and nutrient is of utmost importance to sustain the growing mass of the tumor (11, 15, 19, 20). To combat this hypoxic condition, the cells of the TME activate the hypoxia-inducible factor (HIF-1) signaling (130). The expression of HIF-1α on CD4+ T cells along with different cytokine receptors in MF patients has been associated with disease progression (131). The role of HIF-1 in the regulation of the vascular endothelial growth factor-A (VEGF-A) and, subsequently, in angiogenesis is well established (132). In the MF, the level of angiogenesis is directly linked with tumor progression, which provides suitable conditions in the TME of MF for tumor growth (133). It has been observed by analyzing the expression of CD34 that there are an increased number of microvessels in MF in comparison with normal skin tissue (134, 135). The lesioned skin at different stages of MF, viz., IB, IIB, and IVA1, have higher VEGF-A mRNA levels in correlation with CCL27 mRNA levels (136, 137). Another evidence for the correlation between tumor progression and angiogenic markers came from the finding that overexpression of placental growth factor (PlGF), another member of the VEGF family in stages IIB and IVA1 lesional skin of MF, has been linked with CCL27 and IL-4 expression (136). It has been reported that in stage IIB, the serum PlGF levels are upregulated along with serum CCL17, Ang-2, and IL-10 levels (136). Alternation induced in mitochondrial DNA (mtDNA) due to frequent exposure to UVA radiation results in impairment of mitochondrial function and upregulation in metalloproteinase MMP-1 expression (138, 139). In the case of the advanced MF stage, the overexpression of metalloproteinases MMP-2 and MMP-9 occurs in microvascular endothelial cells along with that in stroma fibroblasts (136, 140, 141). Therefore, it is clear that TCL aptly subverts the signaling pathway including the cytokine-mediated pathway to adapt to TME stress conditions and may be a point of focus to avoid tumor development in CTCL.

### 2.2 Tumor microenvironment in peripheral T-cell lymphomas and cytokine-mediated signaling

The disease pathogenesis of PTCL is quite different from CTL, and it has been noticed that PTCL varieties of TCL can be predicted for their developmental organ site based on special gene expression signatures which are organ-specific (142). This observation indicates the importance of the TME in developing the specific type of tumor (143). As with other tumor cells, the TME of PTCLs constitutes various non-neoplastic cells that are the basis of characterization as in the case of angioimmunoblastic T-cell lymphoma (AITL) in which vascularization patterns and contents of immunoblast are considered as defining features (55, 144). Similarly, in the case of NOS, the follicular variant of PTCLs, the growth pattern of lymphoma cells, and their association with follicular dendritic cells are the distinguished features (145, 146). The lymphoepithelial variants of PTCLs are characterized by the presence of numerous histolytic infiltrates in the TME (142, 147). It has also been observed that the composition and nature of the TME vary from patient to patient in the case of PTCLs (142).

Although the exact nature and interaction between the individual component in the TME of PTCLs are still not well understood, it is clear that the TME influences the tumor progression in the case of PTCLs (55), as in the case of AITL which had been named angioimmunoblastic lymphadenopathy initially but has later been renamed as immunoblastic T-cell lymphomas due to a better understanding of its TME and its intercellular interactions that occur in the TME (148). Initially, it was believed that the reason behind the disease is an abnormal hyperimmune B-cell system but the elucidation of clonal T-cell receptor (TCR) gene rearrangements and identification of clonal cytogenic abnormalities confirmed the neoplastic nature of immunoblastic T-cell lymphoma (49). AITL is unique because in this TCL, neoplastic cells migrate from the lymph node to many different tissues and anatomical sites to develop appropriate conditions to support their growth (149).

**Figure 2:**
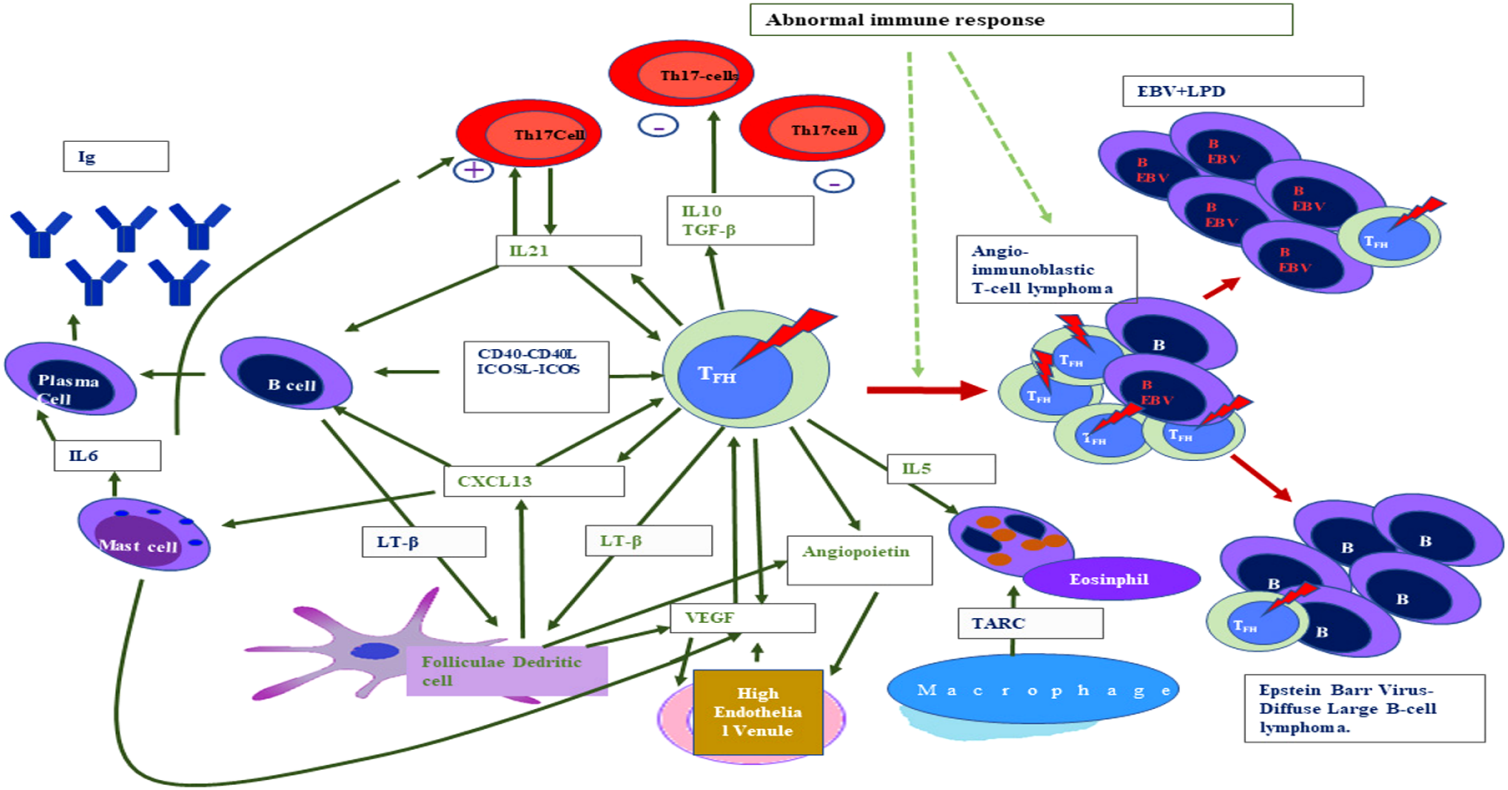
Interaction between different cellular non-neoplastic components of the tumor microenvironment and T-lymphoma cells in the case of angioimmunoblastic T-cell lymphoma (standard abbreviation used).

Flowcytometry analysis has shown that lymphocytosis is uncommon in AITL, but a variable number of immune cells with abnormal immune-surface phenotype occur and are based on the morphology of these cells in the TME (150). The AITL can be referred to as patterns I, II, and, III. Pattern III with the diffuse proliferation of lymphocytes is more frequent than pattern I (hyperplastic follicle pattern) and pattern II (with depleted follicles) (151, 152). The elucidation of the fact that AITL originated from T-follicular helper (Tfh) cells enhances the understanding of the pathophysiology of AITL (145, 146, 149) (**Figure 2**). The basis of this is the expression of the Tfh marker specially CXCL13, which has been further validated by a study of genome-wide transcription markers (145). The role of Tfh cells is critical as its interaction with B cells at the germinal center (GC) is essential for B-cell survival, somatic hypermutation, and immunoglobulin class switching (153). These interactions of Tfh cells with B cells at the germinal center ultimately led to the development of effector plasma cells and memory B cells (58, 153, 154). The expression of chemokines CXCL13 and cytokine IL-21 is very crucial for the recruitment of B cells in the germinal center (155). The expression of CXCR5 (receptor of CXCL13) is responsible for Tfh localization in the GC (156). At the cellular level, tumor cells of AITL vary in size (small to medium) and are mature αβ CD4+ and CD8+ T cells (157, 158). Since these AITL cells are derived from Tfh cells, they continue to express various Tfh-associated proteins like BCL6, ICOS, C-MAF, CD200, CXCL13, and SAP (157, 159). In the AITL neoplastic cells, neutral endo-peptidase CD10 is also observed in variable proportions (160). In conclusion, these observations indicated that CD10 and Tfh markers highlighted the source of origin of this tumor cell which resides among non-neoplastic reactive T cells (CD4+ and CD8+) in the TME of AITL (144, 157, 160). It is a fact that in the TME of AITL, the non-neoplastic cells often outnumbered the neoplastic cells, and clinically, dysregulated inflammatory and immune responses are observed in AITL manifestation instead of tumor growth-related complications, which support the theory of para-neoplastic immunological dysfunction (49).

The molecular profiling of AITL gene expression clearly indicated the influence of the TME, which is evident by overexpression of B cells and follicular dendritic cell (FDC) genes such as various immunoglobulins, clusterins, cytokines, and cytokine receptors along with genes related to the extracellular matrix (161). EBV-infected large B-cell blasts are often found in the TME of the majority of AITL (162). In the TME of AITL, the contact interaction occurs between B cells and Tfh cells and neoplastic T-cell rosettes are generally found around large B-cell blasts (55, 144). In the TME, these interactions occur via the ligand–receptor binding like cytokine binding with its respective receptor on the surface of the interacting cells like CD4-CD40L and ICOS-ICOL (163, 164). The role of Tfh in the TME is very crucial because it secretes different soluble factors such as cytokine which act in an autocrine manner to support TCL (151, 157, 165). CXCL13 is the most crucial immuno-mediator secreted by Tfh which subsequently promotes B-cell expansion along with plasmocytic differentiation, which ultimately leads to abnormal immune manifestations associated with AITL (55, 144–147). This dysimmune function in AITL is also promoted by cytokines released from other cells of the TME (166). For example, IL-6 released from mast cells and neoplastic Tfh cells promote plasmacytosis of IL-21, another soluble factor released from Tfh cells that work in an autocrine fashion and subsequently exert a positive effect on B cells resulting in increased antibody production by plasma cells (58, 150, 151, 153). It has been reported that lymphotoxin β released by B cells after induction by CXCL13 is also expressed in AITL tumor cells (167, 168). This lymphotoxin β  is suspected to be involved in the induction of FDC proliferation, and these FDC of the TME may show an abnormal expression of FDC-specific markers such as CD23 and CD21, but a direct link between abnormal expression of CD21 along with CD-23 with development of FDC neoplasm has not been reported (58, 151, 153, 168). The AITL overexpression of the vascular endothelial growth factor (VEGF) is associated with an increase in the vascularization evident in the disease and immunostaining of neoplastic cells and endothelial cells of the TME, revealing that there is some paracrine or autocrine loop as evident by the expression of both VEGFs to sustain the angiogenesis (49, 157, 158, 161, 163, 166). Since angioprotein-I is also expressed by AITL and FDC, the role of angioprotein-I may also be crucial in increased angiogenesis (157).

Eosinophils are always present in the TME of AITL albeit in differential numbers, as evidenced by blood hypereosinophilia in around 30%–50% of AITL subjects (169). It has also been reported that bone marrow hyperplasia along with eosinophilia is common in AITL (159). The link between eosinophilic infiltrates present in the tumor biopsies and CCL17/TRAC mRNA expression on mononuclear cells present in the TME has been reported (170). It has also been observed that the neoplastic cell of the TME expresses IL-5 but not CCL11/eotoxin1 (171). The CD-3+ T cells isolated from AITL overexpressed IL-4, IL-5, and IL-13 than the normal lymphoma, which may be associated with eosinophilia observed in the AITL subjects (148, 171–173). However, it is still not clear about the role of eosinophilia in the disease pathology of AITL. In an epithelioid variant of AITL, the TME contains macrophages in variable numbers with numerous epithelioid cells in clusters, which creates mistakes in diagnosis and can be misinterpreted as a granulomatous disease (152). The macrophages present in the TME of AITL are of both M1 and M2 phenotypes (2, 174). The role of T-cell mediated response is not clear, but CD8+ T cells are suspected to play an immunosuppressive role; usually, the TME of AITL is immunosuppressive which is evident by the low number of Treg cells together with M2 macrophage expansion and Th17 cell accumulation (142, 148, 152, 175, 176). Cytokines such as IL-10 and TGF- β produced by Tfh inhibit T-cell immune response by suppressing the proper functioning of normal CD4+ T helper cells (177, 178). In the TME, the FDC differentiation is induced by TGF-β in an autocrine manner and this immunosuppression in the TME reactivates EBV, which further induces Tfh-cell and B-cell expansion (179, 180). During the disease progression, the TME cytokine milieu of AITL changes in favor of neoplastic cells with a simultaneous decrease in the proportion of non-neoplastic cells (179). An experiment conducted on a mouse model of NOG in which serial transplantation of AITL had been done ended in the subsequent reduction in an immuno-active components like the B cells and CD8+T cells (161). These observations suggested that the TME microenvironment is dynamic and the neoplastic cell growth becomes independent from the influences of the TME with time. In T-cell lymphoma especially in PTCL, the tumor development is dependent upon the supporting environment of the TME and the alternation in cytokine profile of immune cells and the TME is interdependent, as evidenced by the various studies discussed in this section especially in the context of PTCL.

## 3 Alternation in cell signaling and subsequent reprogramming of T-cell lymphoma metabolism

As reviewed in the previous section, the role of various signaling pathways in the alternation of the TME especially cytokine-mediated signaling is crucial and these alternations result in the hypoxic condition and acidic environment in the TME, which is the hallmark of any cancer (11, 18). To overcome this hostile microenvironment, the TME normalizes oxygen level and ensures adequate nutrient supply by promoting angiogenesis through co-ordination between its various intrinsic and extrinsic factors (33, 34). The upregulation of aerobic glycolysis in cancer cells is one example of an intrinsic factor that resulted in the “Warburg effect”. The “Warburg effect” results in the upregulation of glycolysis along with enhanced lactate production even in inadequate availability of oxygen (11, 13, 15). This increased level of lactate, in turn, alters the extrinsic factors resulting in alteration in the extracellular matrix (ECM) and angiogenesis along with tumor invasion (18). It has been shown that oncogenes like C-Myc, KRAS, and PI3K regulated the uplifting of glycolysis rate by upregulating the expression of various proteins of the glycolytic pathway (32–34).

These oncogenes are also responsible for increased glutaminolysis, a hallmark of tumor metabolism which resulted in both ATP generation and synthesis of precursors for the biosynthesis of other macromolecules such as nucleic acid, proteins, and lipids (11, 18). Previously, due to misinterpretation of the “Warburg effect”, it had been postulated that in cancer cells, the mitochondrial oxidative phosphorylation gets defective but now the importance of the mitochondrial electron transport chain in tumor progression has been accepted as evident by intra-aperitive 13C tracing experiments in human suffering from brain and lung cancer (32, 33, 37, 181). In various cancer types, enzyme mutation is another important intrinsic factor (15). In acute myeloid leukemia, lower-level gliomas and secondary glioblastomas are associated with somatic mutation of isocitrate dehydrogenase-1 and -2 (IDH1 and IDH2), which results in the conversion of alpha-ketoglutarate (αKG) to D-2-hydroxyglutarate (D-2HG), subsequently altering the various alpha-KG-dependent deoxygenase (11, 13, 15, 182). In addition to that, tumor progression is supported by a mutation in either succinate dehydrogenase complex (SDH) or fumarate hydratase (FH) enzymes which have antitumor effects in hereditary tumor syndromes (183–185). Fumarate also triggers cell migration in endometrial cancer (185).

Naive T cells are metabolically similar to other cells and need IL-7 for the maintenance of their basal required metabolism (19). The naïve T cells are smaller in size and generally do not divide. Metabolically, they are highly oxidative and depend completely on the efficient metabolic breakdown of glucose and glutamine (13, 19, 20). In humans, these naive T cells have a comparatively longer life span ranging from months to years. This longer life of T cells is attributed to the continuous induction of IL-7 (22) and alteration in anti-apoptotic protein BCL-2 (B-cell lymphoma-2) which result in the prevention of apoptosis initiation. Upon activation, the metabolic portfolio of a T cell gets a very significant transformation (19, 20, 24). Once they are activated through the TCR and co-stimulatory signal, T cells require continuous IL-2-mediated signaling to maintain their activated state (38). All these signaling mechanisms like TCR-mediated, CD-28-mediated, or IL-2-mediated trigger different pathways to support the upregulated metabolism (19, 24, 38). To support the highly activated and proliferative state, the activated T cells increase the glucose uptake, and upon activation, changes in calcium flux and lactate production occur instantly in these immune cells (19, 24).

Although TCR signaling is needed for initiation of upregulation in glucose transportation, for maximum glucose uptake, CD-28-mediated signaling along with AKT pathway initiation is a must (186, 187). IL-2 produced by activated T cells maintains this upregulation in glucose transformation in an autocrine fashion (188–190). This increased uptake of glucose is accompanied by upregulated oxygen consumption, which is quite similar to the cancer cells (18, 19, 33, 188). Increased glucose uptake results in ATP generation through glycolysis as well as the production of precursors of nucleic acid, lipids, and antioxidants through the pentose phosphate pathway (PPP) (15, 20). These metabolic changes are dependent on cytokine stimulators (191). It has been reported that T regulatory cells are less glucose-dependent metabolically, and rather than this, they depend on fatty acid oxidation to support their metabolism (192, 193). It has been demonstrated that the naive cells activated without glucose do not develop into effectors cells but are easily differentiated into T cells (19). Recently, the role of hypoxia-inducible factor (HIF-1) in the development of Th-17 cells has been documented (194, 195). This underlying difference in metabolism between effector T cells and T-reg cells is very crucial to understanding TCL metabolism in the context of normal cell metabolism (15, 38). JAK-STAT and P13K-AKT downstream of the cytokine signaling pathway of T-cell activation are very crucial to maintaining the growth and proliferation of activated T cells (19, 191, 196). STAT is associated with the IL-2 receptor, and upon IL-2R binding with IL-2, it upregulates some proteins such as BCL-2, C-Myc, and cyclin D (188, 189). The P13K-AKT pathway is associated with the CD-28 co-stimulatory receptor and cytokine receptor in almost all cancer cells including TCL, and this pathway is activated in an inappropriate way (22, 38, 191). Phosphorylated AKT in TCL activates the mammalian target of rapamycin complex-1 (mTORC-1) to bring upregulation in protein translocation (197). The activated mTORC-1 deactivates protein synthesis inhibitor eIF4E binding protein to allow cap-dependent protein synthesis; furthermore, mTORC-I activates S-6 kinase resulting in Sb-ribosomal protein activation (198). The availability of free amino acid is critical to continuing the activity of mTORC-I, and this continued activation of mTORC-I ultimately results in the overrepresentation of glucose transporter-I (Glut-I) along with C-Myc and HIF-I alpha in TCL cells (199). The activation of Akt downstream to CD28 maintains the Glut-I expression on the cell surface and also helps in the upregulation of hexokinases for maximum utilization of glucose by the cell along with the activation of PPP to facilitate the synthesis of other macromolecule precursors (15, 20, 196, 200). This metabolic phenotype of activated T cells is different from other normal cells but similar to the TCL cells (93).

Mitochondrial membrane permeabilization is a crucial step to initiating apoptosis, and Akt activation through cytokine signaling has the ability of hexokinase localization which may hinder mitochondrial membrane permeabilization (201, 202). It has also been reported that activated Akt promotes ubiquitination of tumor-suppressive P^53^ and subsequent degradation of P^53^, which results in suppression of synthesis of the proapoptotic P^53^-upregulated mediator of apoptosis (PUMA) protein (203, 204). A significant decrease in Puma accumulation inside the activated T cell, as well as T lymphoma cell, is associated with upregulation in glucose uptake and activation of Akt (201, 204). The changes in metabolic phenotype brought by upregulation in glucose uptake are also associated with the stability of anti-apoptotic protein myeloid cell leukemia-I inside the activated T cell and T lymphoma cell (205). This observation indicates that Akt and other signaling pathways inside activated T cells may result in anti-apoptotic signaling alternation (93).

**Figure 3:**
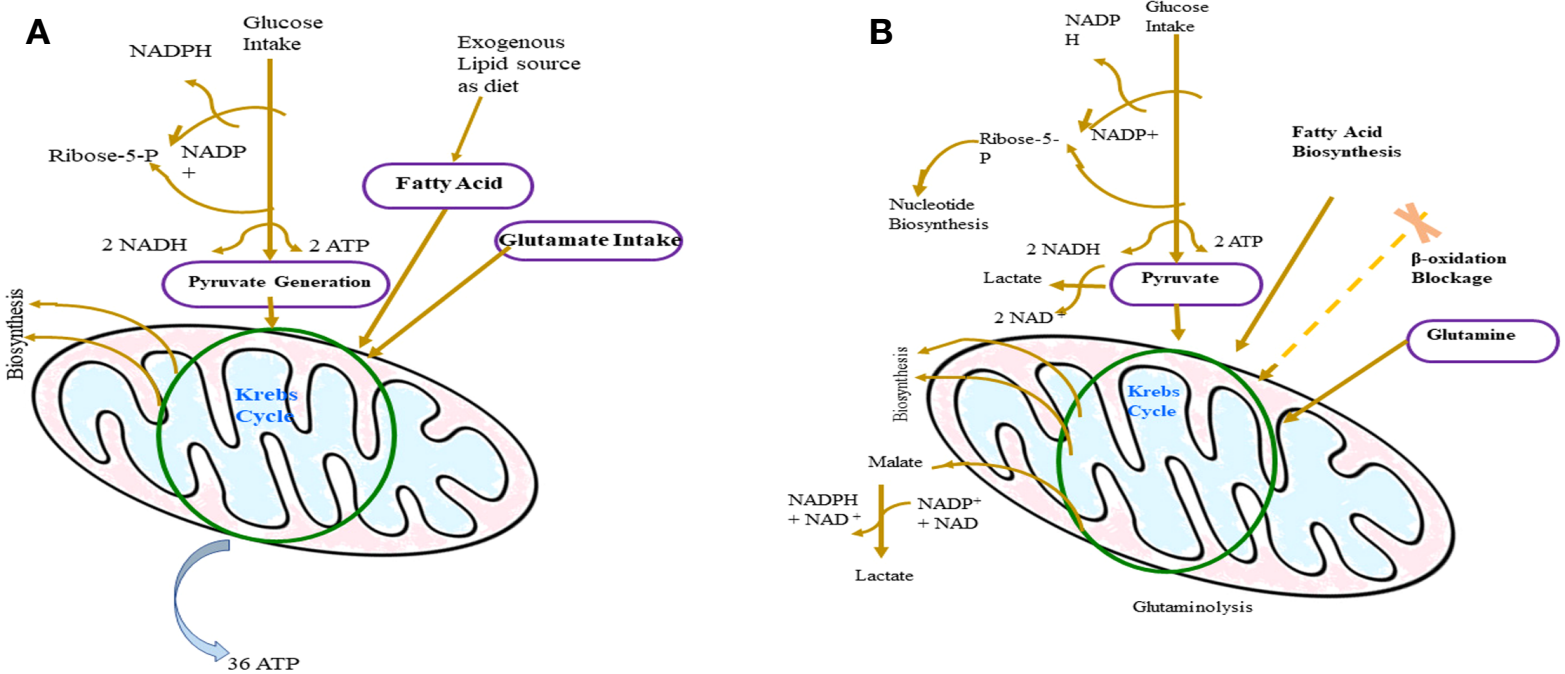
Difference between the metabolism of normal cells **(A)** and T-lymphoma cell **(B)**. In T-lymphoma cells, glucose is the backbone of its growth as it fuels the biosynthesis of other important molecules like protein and nucleic acid. Glutamine is the main source of lactate production in tumor cells and in addition to that glutamine supports tumor growth by synthesizing Kreb’s cycle and NADPH regeneration intermediates.

As discussed above, the metabolic alternation observed in the activated T cells is very similar to T lymphoma cells (**Figure 3**). It has been reported that many cancer cells including TCL upregulate the expression of the embryonic M2 isoform of pyruvate kinase (PKM 2) which results in the promotion of aerobic glycolysis and lactate production, bypassing the TCA cycle (206). An irregular expression of C-MYC or HIF-I alpha in tumorigenic T cells inactivates pyruvate dehydrogenase and upregulates lactate dehydrogenase A (LDHA), which promote the conversion of pyruvate to lactate (194). Recently, it has been reported that tumor cells like many activated lymphocytes use extracellular glutamine as a carbon source as well as a primary energy source, and this is linked to the inappropriate expression of C-MYC in mice (93, 207). Although T lymphoma cells are very similar to activated T cells, tumor cells have mutations in enzymes of the TCA cycle which is not the case with normal activated T lymphocytes (93). Examples are the mutations observed in isocitrate dehydrogenase (IDH) I and IDH 2 in gliomas and leukemias (208, 209). IDH is an important enzyme that regulates the conversion of isocitrate to alpha-keto-glutarate, and after mutation, they convert alpha-ketoglutarate to 2-hydroxyglutarate, a novel metabolite which subsequently alters the epigenome of the cell (208, 210).

The T lymphoma cell expresses muted versions of growth factor receptors and thus mimic the activated lymphocyte for their survival (93, 211). This is evident by the observation that in more than 50% of patients with T-cell acute lymphoblastic leukemia, proteins of the Notch pathway were mutated (212). The Notch pathway is very critical for the development and lineage selection of lymphocytes (213). As discussed, the expression of C-Myc is associated with upregulated glucose metabolism and mutations resulting in the overexpression of C-Myc have been reported in various lymphomas like Burkitt’s lymphoma (196). It can be concluded that these alternations in the signaling pattern of cancer cells including T lymphoma cells are the chief reason behind the Warburg phenotype and constitutive expression of Akt, along with C-Myc results in upregulation of aerobic glycolysis, inhibition of the TCA cycle (through the activation of proto-oncogene), and suppression of apoptotic genes. It has been clear that the metabolic reprogramming of tumor cells is essential for their survival and development. As discussed in this section, T-lymphoma cells mimic activated T cells metabolically, but the T-lymphoma cell differs from normally activated T cells because it expresses various oncogenes and inhibits tumor-suppressor genes. Due to this difference, whereas normally activated T cells either go through apoptosis or attain the state of anergy, T-lymphoma cells continue to survive and divide. To support this continuous growth, metabolic reprogramming is essential, which is also termed as the “Warburg effect”. For the effectiveness of the Warburg effect, signaling mediated by various cytokines is essential, as highlighted in this section. Various signaling pathways downstream of cytokine receptor-mediated signaling pathways like P13K-AKT and JAK-STAT are responsible for the pro-tumorigenic metabolic alternation. Cytokines like IL-7 and IL-2 have proven roles in the maintenance of basal metabolic requirements, and as discussed, their expression level is directly linked with metabolic reprogramming. Therefore, on the basis of the findings discussed in this section, it may be easily concluded that cell signaling alternations especially cytokine mediated are crucial for T-lymphoma cells’ metabolic reprogramming.

## 4 Cell signaling and cytokine in the context of T-cell lymphoma development

**Figure 4:**
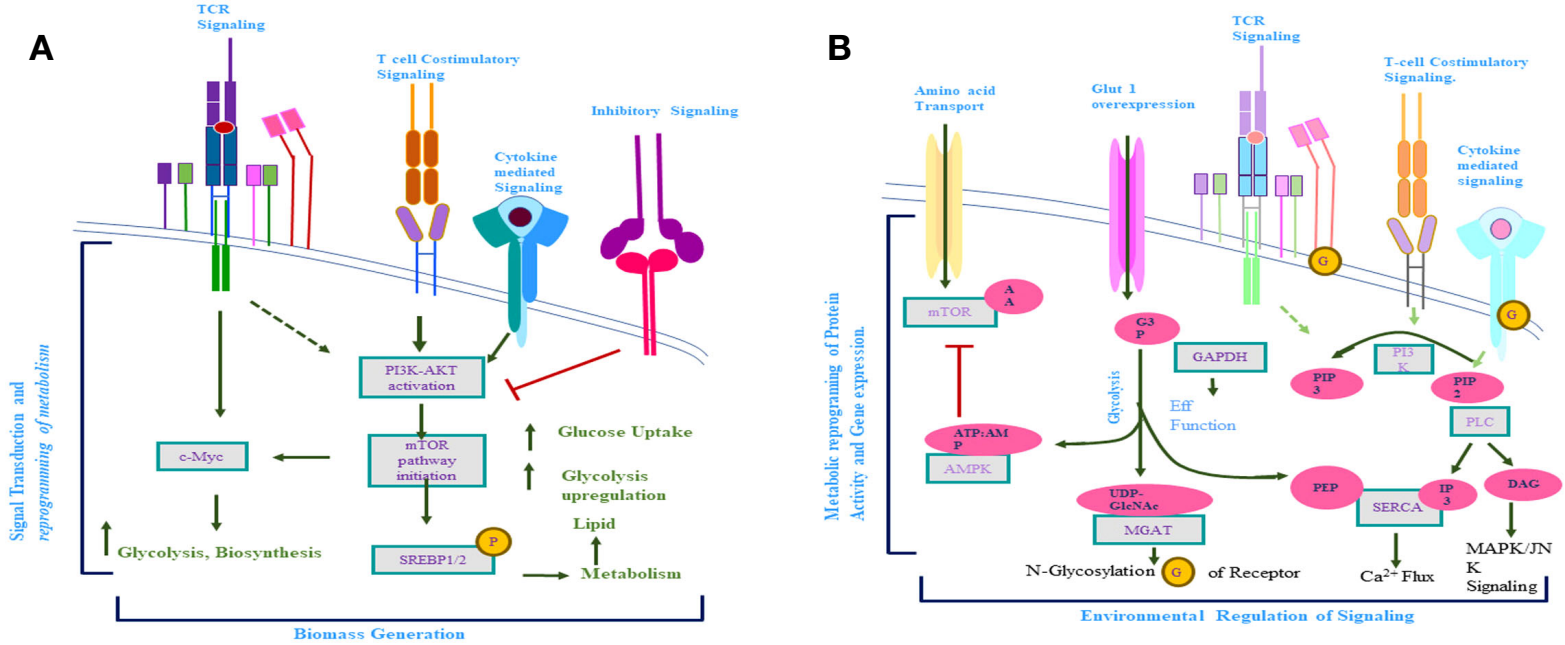
**(A)** Activation of T-cell receptor (TCR)-mediated signaling induction of costimulatory and cytokine receptor-mediated signaling takes place. Activation of these signaling pathways results in the downstream activation of mTOR and C-Myc and the reprogramming of activated T-cell metabolism. **(B)** Metabolite produced inside the cell regulates the signaling pathway of activated T cells. Afterward, the activation-activated T cells increase the uptake of glucose and amino acids, which results in a significant increase in the metabolite concern with these biomolecules. This increased level of metabolites influences the signaling pathways (TCR mediated, costimulatory, cytokine mediated) of activated T cells, which further alter the metabolism of activated T cells.

In the earlier section of this review, it has been discussed that the role of the cytokine in the TME development of the T-lymphoma cell and subsequent metabolic reprogramming to support the demanding metabolic requirement is crucial. The recent whole-exome signaling and gene expression profiling studies have shown that in T-cell lymphomas, all three signals (TCR signaling, co-stimulating signaling, and cytokine signaling) get altered (214, 215). In signal-1 in normal T cells, their T-cell receptor recognizes MHC-bound protein which subsequently activates the T cell through the downstream signaling via CD3 (186, 187). This interaction of TCR with the MHC-bonded peptide is very specific and sensitive. Mainly the kinases like LCK (lymphocyte-specific protein tyrosine kinase) and FYN are involved in the autophosphorylation of conserved immune-receptor tyrosine-based activation motifs (ITAMs) located in the cytoplasmic domain of the CD3 (216). These T-cell activation kinase domains get autophosphorylation by the SRC family kinase (SKFs), and dephosphorylation is mediated through several phosphates like phosphates of cytosol, protein tyrosine phosphatase non-receptor-6 (PTPN-6), PPTN22, CD45, and DUSP22 (dual-specificity phosphatase22) (216, 217). The inhibitory signal to the activation of this pathway is mediated by inhibitory C-terminal tyrosine which gets phosphorylated by CSK (C-terminal SRC kinase) and dephosphorylated by CD45 (217). LCK remains continuously active to maintain the signal required for the survival of naïve T cells (186). LCK activity along with ζ-chain activation via phosphorylation recruits ZAP70 (zeta-chain-associated protein kinase 70) and SYK family kinase, which results in the attainment of ZAP70 to its active conformation. This activation of ZAP70 results in the phosphorylation of adopter proteins like LAT (linker for activation of T cells) and SLP-76 (an SH2 domain-containing leucocyte protein of 76 kDa (186, 216) (**Figure 4**). Thus, TCR-dependent signaling of T-cell activation is rather complex involving multiprotein signalosomes, which result in the activation of multiple signaling networks essential for T-cell activation. TCR expression on T lymphoma cells is intact with some exceptions (218, 219). It has been reported that in peripheral T-cell lymphomas not otherwise specified (PTCL, NOS), the elements of the TCR complex were intact in more than 85% of cases, along with a normal expression of ZAP-70, LAT, and ITK. These observations were also similar for angioimmunoblastic T cells which use this TCR-mediated signaling for their growth and survival (220). The required engagement with MHC/peptide to activate TCR-mediated signaling in T lymphoma cells may be provided by the TME of tumorigenic T cells (53). To support this, it has been observed that immature DCs and macrophage favor the T-cell tumor development *in vitro*, which can be inhibited by MHC-blocking antibodies or clonotypic TCR (108). It has not been clear whether antigen-independent TCR signaling occurs in T-cell lymphoma, but the role of TCR-mediated signaling is established through the study on mouse models. Wang et al. have shown that 100% PTCL is developed in mouse models if the TCR remains intact and functional (108, 221). The importance of TCR signaling in TCL is further supported by studies on the role of antigen drug-associated neo-antigen in the development of T-cell lymphoma (38, 220).

Although the role of CD3-TCR-mediated signaling in the development of T-cell lymphoma is universal, in many subtypes of PTCL, there are various alterations that result in TCR complex independent activation of downstream TCR signaling. The case of systemic anaplastic large cell lymphomas (ALCL), in which NPM-ALK (nucleophosmin-anaplastic lymphoma kinase) translocation fuses the catalytic domain of ALK with the dimerization domain of NPM, results in autophosphorylation of ALK resulting in activation of the pathways which are either redundant with RAS/RAF/ERK and P13K/STAT-3 pathway or complimentary to the JAK3/STAT3 pathway (38, 216, 222, 223). This ALK-dependent pathway may work as a substitute for TCR-independent downstream signaling activation (223). Although the mechanism is still not well elucidated, it has been reported that translocation involving the interferon regulatory factor (IRF)-4-DUSp22 (dual-specificity phosphatase 22) results in low expression of DUSP22 which can lead to the uninhibited activation of LCK and ERK and lead to the sustaining of downstream signaling induced by the TCR complex (224, 225). In approx. 20% of PTCL NOS, a translocation is observed between ITK and ZAP70 homologue SYK (226). This translocation results in the fusion of the pleckstrin and T homologous domain of ITK with the SYK kinase domain resulting in a catalytic fusion protein that can phosphorylate PLC- γ1 , SLP-76, and LAT1 along with endogenous SYK (227). This unique ITK-SYK fusion protein induces SRC family kinase-independent mimics of TCR-mediated signaling cascades (224, 227). This fusion protein is reported to be an oncogenic and transgenic expression, causing lymphoproliferative disorder similar to the human PTCL in mouse models (226, 227). Mutation in the gene-specific TCR signaling has been reported in many T-cell lymphomas. One of the examples is a recurrent mutation in RHOA, a small GTPase protein that has been observed in approx. 70% AITL and around 20% of PTCL and NOS. This RHOA (RAS family protein) binds active GTP, and the mutation (G17V) in the GTP binding site of RHOA in T-cell progenitors present in the thymus results in the development of T-cell lymphoblast leukemia in mouse models (228–231). It has been reported that when dominant-negative RHOA (Gi7V) is transduced in the cell line, it results in altered F-actin stress fiber development (230–232). This actin cytoskeleton is crucial for proper TCR activation since it maintains the spatiotemporal integrity of the cell membrane (233, 234). Other important proteins crucial for regulating the actin cytoskeleton are E2H2 (polycomb group protein) and Vav1 (A guanine exchange factor) (233).

In addition to TCR-mediated signaling, additional non-MHC signaling is essential for the proper activation of T cells. Various co-stimulatory signaling receptors have been reported to belong to either the Ig superfamily or the TNF receptor superfamily (TNRSF) (235, 236). Along with this activator co-stimulatory receptor, there are numerous homologous receptors with inhibitory effects present on T cells enabling the maintenance of fine-tuning of T-cell-mediated immune response (235). These co-stimulatory receptors mediate the activation of different signaling pathways like P13K, MAPK, NFAT, and NF- Kβ (236). It has been observed that CD28 engagement induced the proliferation of Sézary cells (237). Recently, a recurrent mutation in both the intracellular and extracellular domains of the CD28 receptor is reported in some PTCL resulting in increased affinity of receptor CD28 to its corresponding ligands (238). These observations are suggestive of the role of CD28 co-stimulation in T-cell lymphoma pathogenesis. Furthermore, in Sézary syndrome patients, the fusion of the transmembrane and extracellular domains of CTLA-4 with the intracytoplasmic domain of CD28 has been reported, which results in upregulation in CD28 signaling (239). The abundant availability of CD28 ligands in the TME supports the growth and development of T-cell lymphomas (34).

Following the engagement of TCR and CD28, the expression of ICOS (inducible T-cell co-stimulator) was induced. A knockout experiment in mouse models indicated the important role of P13K in ICOS-dependent proliferation, differentiation, and activation of T cells (38, 240). Although ICOS is expressed in various types of T cells, it is present in the germinal center and essential for GC homeostasis (241). It has been reported that AITL which has been originated from Tfh cells highly expresses ICOS (242). Another co-stimulatory receptor is CD134 (ox40) which belongs to TNRSF that is induced after TCR engagement but is not expressed by either naive or memory T cells (243). The ligand of CD134 is expressed by professional antigen-presenting cells, active T cells, and some non-hematopoietic cells (244). After activation through its ligand, CD134 trimerizes and its recruitment in the lipid raft is followed by association with TNFR-associated factor (TRAF) proteins 2, 3, and 5 through a conserved motif present within its cytoplasmic tail (245, 246). CD134 is expressed in more than 95% of AITL, 17% in PTCL, and NOS and absent in anaplastic large cell lymphomas (ALCL) (247). Similarly, TNFRSF8 (CD30) is expressed in a variable manner in PTCL (248). Although the role of TNFRSF in T-cell lymphoma pathogenesis is poorly understood, the importance of TNFSFs in T-cell lymphoma is indicated by the fact that TNFRSFS homologous to B cells plays an important role in B-cell lymphoma pathogenesis (249).

Although MHC-peptide-dependent and co-stimulatory signals are crucial for the activation of naive T cells, cytokine-dependent signaling is essential for effective T-cell response. Various studies reported the important role of IL-12 and IFN-alpha/beta in CD8 T-cell maintenance and activation (38, 250). The signaling pathways of most of the cytokine receptors are induced by respective cytokine binding to its receptor followed by activation of a family of Janus kinases (JAK1, JAK2, JAK3, and tyrosine kinase-2), which is associated with the cytokine receptor cytoplasmic tail. Cytokine binding to its receptor results in conformational changes in JAKs which subsequently phosphorylate signal transducers and activators of transcription (STAT) monomer (38, 251). Activated STAT dimerizes and after translocation to the nucleus regulates the gene expression (251). In T-cell lymphoma, any cytokine that suppresses host anti-tumor activity is considered a tumor-promoting cytokine. It has been reported that various inflammatory and homeostasis cytokines have a role in T-cell lymphoma pathogenesis (38, 87). Transcription factor GATA-3 is a master regulator of Th2 differentiation since it binds to the locus of Th2-specific cytokines. Two dominant subclasses of cytokines have been reported in the case of PTCL: one is GATA-3 enriched (IL-4, IL-5, IL-13), and another is t-bet enriched (IFN-gamma and IFN-gamma inducible gene). In the PTCL, NOS and the GATA-3+ subclass of T-cell lymphomas resembled CTCL on a molecular level in which type 2 cytokines have a direct role in the proliferation and survival of tumor cells. IL-5-dependent hypereosinophilia has been observed in the GATA 3+ subclass of PTCL (64, 252). Recent whole-genome and whole-exome studies have reported that recurrent mutations in JAK and STAT genes are frequent in some classes of T-cell lymphomas (70). For example, in T-cell polympholytic leukemia (T-PLL), recurring mutation in gamma-chain, JAK1, JAK3, and STAT has been reported in more than 75% of cases (253–255). Similarly, STAT5b mutation is frequent in gamma F-PTCL and NK-cell-derived lymphomas (255). Although STAT3 mutation is not very prevalent, it has an important role in indolent T-cell lymphoproliferative disorders (256). It has also been reported that the PTPRK (receptor type tyrosine-protein phosphatase K) gene located at chromosome 6q is mostly deleted in extranodal NK-cell lymphoma and T-cell lymphoma, which results in STAT3 dephosphorylation and subsequent activation of STAT3 in approx. 50% of NK/T-cell lymphoma (257). It has been observed that JAK/STAT mutation is more prevalent in TCL than recurring mutation in its cytokine receptors (258). Mutation in chemokine receptor CCR4 is an exception, which is more frequent in TCL (259). The c-terminal truncating recurring mutation in CCR4 is reported in more than 25% of ATLL cases (260). This mutation in CCR4 hinders its internalization, a kind of gain-of-function mutation, and it was reported to be associated with increased PI3K/Akt activation which in turn plays a crucial role in metabolic reprogramming in TCL (260, 261).

It is easy to describe the role of these different T-cell signaling pathways in TCL pathogenesis in isolation, but in actuality, all these signaling pathways are interwoven and regulate the TCL pathogenesis in a coordinated manner. The cross-talk between these signaling receptors has been observed, and their dependency on each other in TCL development and progression has been reported. However, it is a fact that alternation in TCR-mediated signaling is crucial to bringing required changes in other receptor signaling pathways for the survival of T-lymphoma cells. Through the earlier works discussed in this section, it has been clear that in addition to metabolic alternation, cytokine-mediated signaling is also important for the overall development of T-lymphoma cells. Signaling mediated by these cytokines regulates the different stages of development, i.e., from naive T-cell to activated T-cell. In fact, all three T-cell signaling pathways, i.e., TCR mediated, co-stimulatory, and cytokine-mediated, play a crucial role in TCL, which helps in the differentiation of T-lymphoma cells to attain its tumorigenic morphology and physiology. Cytokine-mediated signaling depends upon the various intrinsic and extrinsic factors, and it may be easily concluded that cytokine plays an important role in deciding the fate of the developing T-cell lymphoma in the tumor microenvironment.

## 5 Interrelation of cytokine and metabolism in T-cell lymphoma

**Figure 5:**
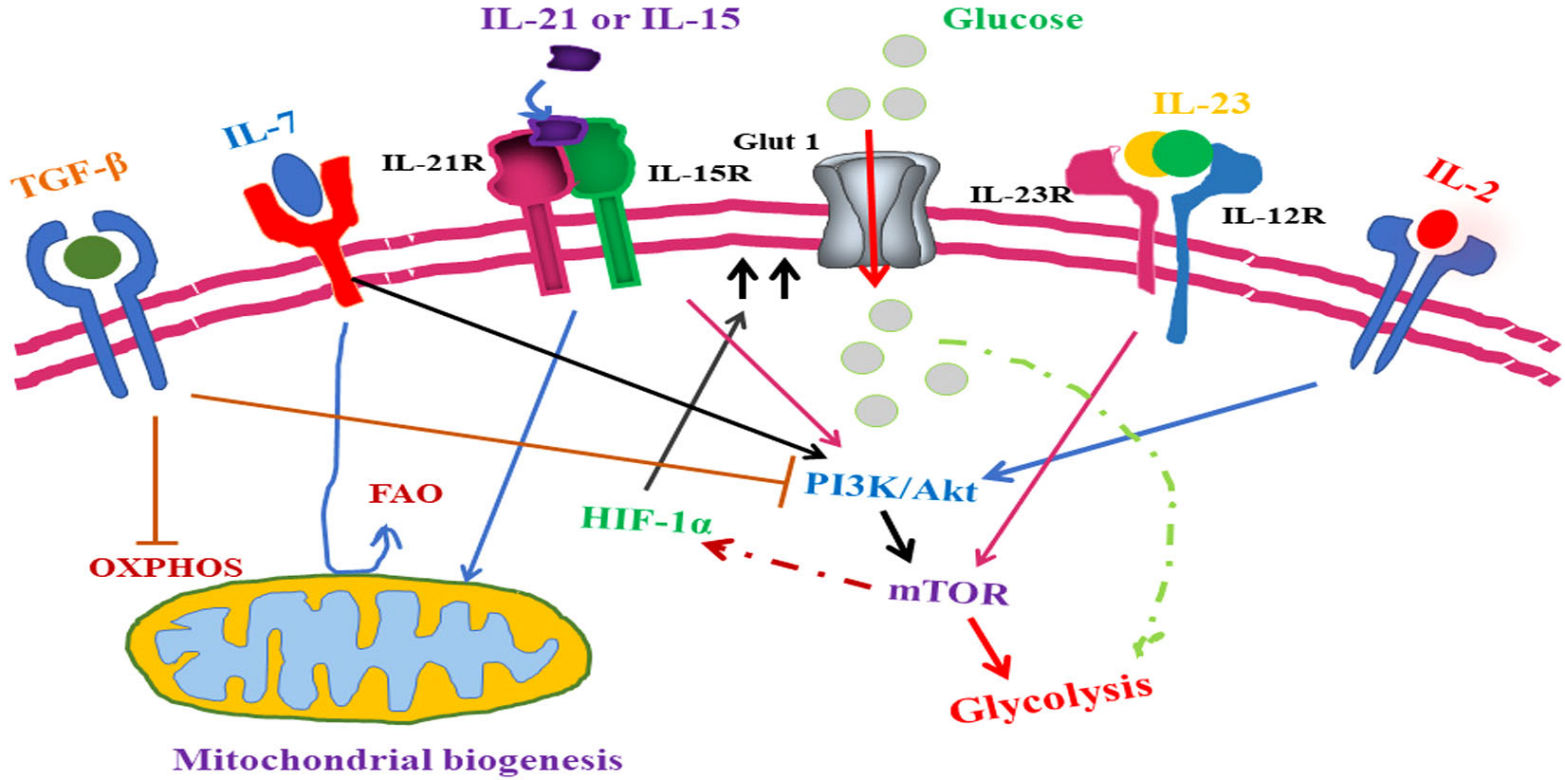
Role of cytokine-mediated signaling in T-cell lymphoma metabolic reprogramming. TGF-β, an anti-inflammatory cytokine, inhibits T lymphoma cell glycolysis whereas IL-7 signaling promotes glycolysis through upregulation of Glut1 expression. With a similar role in antioxidant production, IL-15- and IL-21-mediated signaling plays an important role in oxidative phosphorylation along with mitochondrial biogenesis inside the T-lymphoma cells. IL-15- and IL-21-mediated signaling induces the mTOR pathway and subsequent alternation of T-lymphoma cell metabolism. In addition to promoting the growth of Th17 helper cells, IL-1β also induces the mTOR pathway. Although IL-2 promotes mTOR activity in both CD4+T cell and CD8+ T cell through different pathways, in CD8+ T cell, IL-2 induces mTOR through PDK whereas in CD4+ T cell, IL-2 induces mTOR through activation of the PI3/Akt pathway. Upregulation in amino acid intake by T-cell lymphoma cells is promoted through overexpression of SLC7A5.

Metabolism and immune response are interlinked, and as discussed in earlier sections, metabolic alternation is the hallmark of the tumor cell and this metabolic reprogramming in mammalian immune cells is a crucial immunological adaption to mounting a proper immune response (22). This association of metabolism with immune response has been known for the last 60 years (19, 23) (**Figure 5**). It has been reported that IL-6, a pro-inflammatory cytokine, can regulate glucose as well as lipid metabolism (262). It is a fact that increased uptake of amino acid is crucial to support continuous mTOR activity and, along with C-Myc expression, IL-2 signaling plays a significant role in this (191, 198). It has been reported that IL-2 signaling induces amino acid transporter SLC7A5 and it redirects T-cell metabolic reprogramming through pyruvate dehydrogenase kinase (PDK)-mediated upregulation of mTOR, which does not require P13K/AKT signaling in CD8+ T cells (191, 198, 263). The role of IL-2 in Treg-cell metabolism regulation is evident by the high expression of the IL-2 receptor (IL-2R), which is accompanied by the phenotypic and functionally active state of Treg cells (189, 264). IL-2-mediated signaling is crucial for T-cell metabolism as it co-ordinates P13K/AKT/mTOR activity, upregulates SLC7A5 expression, and upregulates C-Myc expression in CD4+T cells and CD8+ T cells (60, 189, 198). To support functionally active Treg cell metabolism, a continuous level of glycolysis and lipid biosynthesis along with the proper mitochondrial function is required and IL-2 has been reported to play an important role in all of these metabolic requirements of Treg cells (198, 265, 266). Another important cytokine for the maintenance of T-cell metabolic homeostasis is IL-7. It has been reported that IL-7-mediated signaling plays a crucial role in sustaining glycolysis in T cells through STAT-5-mediated AKT induction. It also induces GLUT1 expression in CD8+ memory T cells and IL-7 involved in triglyceride synthesis through increasing the rate of glycol uptake (198, 267).

IL-15 is essential for memory T-cell development, which alters the metabolism of these developing cells through the remodeling of cristae, a fusion of mitochondria, and upregulation in mitochondrial spare respiratory capacity (SRC) (268). IL-15 also supports fatty acid oxidation (FAO) through the upregulation of carnitine O-palmitoyltransferase-1 (CPT1a) which facilitate the transfer of the acyl group of long-chain fatty acyl-CoA from coenzyme A to 1-carnitine. It has been reported that IFN-α, which is an inducer of IL-15, can further upregulate transcription of antioxidant molecules like glutathione reductase, thioredoxin reductase, superoxide dismutase, and peroxiredoxin resulting in the long life of these memory cells (189, 191, 268, 269). This effect of IL-15 may be due to the neutralization of a high level of RO generated through the hyperoxidative phosphorylation activity of mitochondria by these antioxidant molecules. IL-15 plays an important role in the development of the tissue-resident population of T cells by promoting the mTOR activity in memory CD8+ T cells resulting in fastened activation and proliferation of CD8+ T cells in case of reinfection (191, 199, 268, 270). It has been reported that IL-21 has an important role in T-cell metabolism since it promotes fatty acid oxidation, antioxidant production, and mitochondrial biogenesis. It has also been reported that IL-21 has a role in promoting mTOR activity in Treg cells. It has been shown that priming of T cells with IL-21 and IL-15 before using in cancer T-cell therapy prolongs the life of these therapeutic T cells in a cancer mouse model (271–274). Studies have reported that by upregulating mitochondrial activity and reduction in oxidative stress in T cells, both IL-15 and IL-21 delayed the expression of apoptosis markers such as PD1 and LAG-3 (274, 275).

Although the role of inflammatory cytokines in T-cell metabolism has not been investigated much, evidence is growing which is suggestive of the roles of inflammatory cytokines in the alteration of T-cell metabolism (191) (**Table 2**). A study has shown that IFN-γ can sustain the production of effector CD8+ T cells despite of a weak TCR signal (301). This effect of IFN-γ is through the activation of mTOR via STAT-1. The roles of IL-1β and IL-23 are crucial for the shifting of the Treg/Th17 axis toward Th17 through the promotion of human Th17 CD4+T cells. IL-1β-mediated signaling triggers HIF-1α expression along with upregulation of glycolysis-related genes like GLUT1 and HK2 (302–304). This further shift analysis suggested that IL-1β upregulates Th17 cell glycolysis. IL-23 has a synergistic effect on Th-17 cell differentiation and IL-23 is considered as the chief architect behind the Th17 cell phenotype (303). There are various phosphorylation targets on IL-23 identified through phosphor-proteomics analysis including an important glycolytic enzyme PKM2 whose upregulation results in hyperphosphorylation of STAT3 resulting in metabolic alternations (304, 305). It has also been reported that PKM2 is crucial for the homeostasis of the metabolism of Th17 cells (304). It has been observed that these effects of IL-23 can be inhibited by CD28 signaling, which also upregulates SIGIR expression in differentiating Th17 cells, resulting in the limitation of mTOR signaling (306, 307).

**Table 2:** List of Cytokines crucial in T-cell lymphoma pathogenesis and metabolism (Source reference: 87, 191).

Cytokine	Profile	Signaling pathway	Role	References
IL-2	Th1	JAK/STAT, PI3K/mTOR, MAPK/ERK	• Induce T lymphoma cell proliferation and survival • Induction of FoxP3+ Treg cells through STAT5 • Immune evasion	(60, 189, 237)
IL-4	Th2	JAK/STAT	• Induce T lymphoma cell proliferation. • Induction of Th2 predominance and immune abnormalities • Induces secretion of Th1 chemokines	(40, 43, 87, 276)
IFN- γ	Th1	JAK/STAT	• Downregulated in advanced stages of T-cell lymphoma associated with immunosuppression and tilting toward the development of the Th2 inflammatory TME	(40, 191)
IL-7		STAT5	• Continuous support to glycolysis in all CD3+ T cells through STAT5- signaling-induced Akt activation • Overexpression of Glut1 on Tscm CD8+ T cells • Induces upregulation in glycerol intake for triglyceride biosynthesis in CD8+ memory T cells	(267, 277)
IL-8	P Pro-inflammatory	NF-κ β	• Have a role in non-histaminergic itch • Induces neutrophil chemotaxis • Disturb development of infiltrated monocytes into mature DCs • Hampers Th1 responses	(278, 279)
IL-10	Th2	NF-κ β	• Immunosuppression • Induces M2 polarization of TAMs • Induces tumor growth • Promotes IFN-γ production	(39, 40, 280)
IL-12	Th1	JAK/STAT(STAT4)	• Triggers cytotoxic lymphocytes • Downregulation is linked with stage-related abnormalities in cell-mediated immunity and the release of Th1 cytokine	(39, 40, 281, 282)
IL-13	Th2	STAT6	• Hampers the generation of antibacterial protein and induces cutaneous bacterial pathogenesis • Induces Th2 phenotype in cutaneous T-cell lymphoma cells and non-neoplastic T cells • Induces tumorigenic transformation of normal T cells	(283, 284)
IL-15	Pro-inflammatory	STAT3	• Upregulation in FoxP3 expression • Immune response alteration	(38, 268, 269)
IL-17A	Pro-inflammatory	STAT3	• Stimulates angiogenesis • Promotes metastasis in CTCL via induction of IL-22/CCL20/CCR6 axis	(40, 43, 49, 285, 286)
IL-22	Pro-inflammatory	STAT3	• Induction of epidermal hyperplasia via STAT3/CCL20 axis	(287, 288)
IL-23			• Induces mTOR in Th17 cells • Upregulate PKM2 phosphorylation • Regulate metabolic reprogramming in Th17 cells along with IL-1 β	(289)
IL-31	Th2	JAK1/2-STAT RAS/ERK PI3K/Akt MAPK	• Associated with advanced CTCL-linked non-histaminergic itch • Support CTCL tumor growth in an Autocrine fashion.	(43, 290, 291)
IL-32	Pro-inflammatory	NF-κ β	• Induces cell proliferation and survival. • Overexpression of genes associated with survival	(292, 293)
IL-1 β		mTOR	• Induces mTOR and HIF-1 α and reprogramme glycolysis in all CD4+ T cells along with Th17/iTReg	(191, 294)
TNF- α		TRAFs/NF-κ β SAP/ERK	• Has a possible role in the overexpression of metabolism-related genes like HK2, PKM2, and TNFAIP3 in CD4+ T cells especially in memory T cells	(235, 236, 295–297)
TGF- β		Smad signaling and p38 MAPK	• Downregulate mTOR activity inside CD4+ T cells through Smad3-mediated hindrance of PI3K/Akt • Support mitochondrial capacity increase and downregulate glycolysis in T Regulatory cells • Downregulate mitochondrial complex V activity of memory CD4+ T cells	(298–300)

The TNF-α-mediated signaling inhibits CD4+ T-cell maturation and supports those cells which express anti-inflammatory cytokine IL-10 (308). Various studies have shown the role of TNF-α in calcium signaling which control T-cell metabolism. There is a recent report on the impact of TNF-α on the metabolic profile of CD4+ T-cells, but further studies are needed to fully elucidate the role of TNF-α in cellular metabolism in general and of T cells in particular (308–310). Although IL-10 has been reported to have a role in B-cell and macrophage metabolism alternation, evidence for the role of this anti-inflammatory cytokine in T-cell metabolism is few (280). TGF-β induced Smad-3-mediated inhibition of PI3K/Akt signaling in CD4+T cells, which results in the downregulation of important components of glycolysis like GIUT1 and HK2 (280, 298). Due to its suppressive effect on mTOR signaling, TGF-β favors T cells toward the inducible Treg phenotype. A study has reported that circulating Treg treated with TGF-β reduced the suppressive capacity of these Tregs through inhibition of glycolysis via the PI3K/AKT mTOR pathway (311). One *in vitro* study has shown that the presence of TGF-β in the tumor microenvironment can reduce OXPHOS in effector CD4+ T cells (299). Although there is growing evidence that cytokines play an important role in the reprogramming of T-cell metabolism, in turn, this metabolic alternation changes the cytokine profile of T-lymphoma cells as well as of the TME. Further studies are required to understand the mechanism behind cytokine-mediated metabolic reprogramming. As reported and summarized in this section, the cytokine-mediated signaling is important for metabolic reprogramming, and in turn, this metabolic alternation further changes the cytokine milieu of the tumor cell microenvironment in favor of T-lymphoma cell development and survival.

## 6 Future investigation and challenges to make cytokine a diagnostic marker

This is a matter of fact that cytokine receptor-mediated signaling is very crucial to the development and survival of tumor cells. With the advancement in tools and techniques, various novel biomarkers have been identified for the early diagnosis and prognosis of cancer. These biomarkers are a different class of biomolecules, and usually, cancer biomarkers are the molecules derived from the tumor cell and an ideal cancer biomarker should help in screening, prognosis, and cancer stage prediction as well as monitoring of treatment efficacy (312, 313). Another important criterion to be a good biomarker is that techniques based upon these biomarkers should be easy to handle, cost-effective, and reproducible (313). The currently approved biomarker for cancer has limited utility due to its low specificity and sensitivity (314). As cytokines have been associated with every stage of cancer development, these signaling mediators have great potential as a cancer biomarker (315, 316). Usually, a low amount of cytokines exists locally, but the upregulation in the cytokine level is observed in various abnormal conditions including tumors. The potential of cytokine as a biomarker is good because in tumors, the level of cytokines is related with tumor progression and changes in their expression level can be easily detected in body fluid mostly in the blood (317). The level of cytokines such as IL-6, IL-10, IL-8, TNF- α , and VEGF can be co-related with disease progression in ovarian, breast, and cervical carcinoma (317, 318). As discussed in the various sections of this review, the level of cytokine is clearly associated with T-lymphoma progression and metabolic rewiring required for the survival of T-lymphoma cells. As mentioned in **Tables 1**, **2**, various cytokines and their expression level influence the metabolic alternation in T-lymphoma cells. Some more obvious cytokines such as IL-2, IL-6, IL-7, and IL-15 have a direct link with T-cell lymphoma progression, and these cytokines’ expression levels have the potential to be used as biomarkers especially to predict the developmental stage of T-lymphoma tumor cells.

Despite of this clear co-relation, the main hindrance in using cytokines as a biomarker is that cytokines are mechanism specific, not tumor specific. In recent times, numerous works have been reported which evaluated the potential of cytokines as a cancer biomarker, but mostly, these studies evaluate cytokines in isolation; however, in reality, the cytokine expression depends upon various interwoven factors (319–322). The potential of cytokine as an independent biomarker will be realized through the elucidation of the interwoven factors affecting the cytokine level in specific conditions. Another problem in using cytokine as a biomarker is to decide the cutoff value, which is essential to using any biomarker as a diagnostic tool. In the case of cytokine, it is really a huge challenge to differentiate between normal levels and abnormal levels of cytokine because only few studies have been done under any particular condition to decide the base-level value of any particular cytokine to use as a biomarker for any cancer including T-cell lymphoma. Due to the advent of multiplex arrays nowadays, it is possible to monitor the level of different cytokines simultaneously and then use these cytokines as a biomarker group. The receiver operating characteristics analysis (ROC analysis) which is based upon the plot of the true positive rate versus false positive rate of a particular disease at various cutoff may be helpful in deciding the cutoff value of cytokine to enable them to be used as a biomarker (323). In addition, in infectious disease where the roles of cytokines are more clear, the diagnostic potential of cytokines has been established in certain chronic conditions like Alzheimer’s disease (AD) and tumors. A recent study conducted on Malaysian people reveals the clear-cut link between blood IP-10 (interferon gamma inducible protein 10) along with IL-13. The level of these anti-inflammatory mediators was found to be on the lower side in AD subjects in comparison with healthy control significantly (324). Similarly, cytokines such as IL-6 and IL-8 are associated with poor neurological outcomes in individuals who experienced cardiac arrest. The cutoff value to predict poor neurological progress is 1423 pg/ml for IL-8 and 2708 pg/ml for IL-6 with 100% sensitivity and 86% specificity (325). The 1.97-pg/ml serum level of IL-6 with 81.8% sensitivity and 66.7% specificity is found to be the optimal diagnostic cutoff for gastric cancer (326). Two cutoff values, i.e., 400 pg/ml (92% sensitivity and 60% specificity) and 1,200 pg/ml (84% sensitivity and 87%), for IL-6 has been evaluated for stage I and stage II ovarian cancer subjects. The VEGF diagnostic cutoff was found to be 90% sensitive and 80% specific at the serum level value of 400 pg/ml (327). Many studies have been reported with a moderate rate of success to establish the cytokine as a diagnostic or prognostic biomarker. It is clear that to establish the cytokine as a biomarker for T-cell lymphoma development, a particular cutoff value is needed to be identified, and for this, elucidation of complex cytokine biology is needed. The ROC analysis will help in this regard, but the sample size needs to be sufficiently high to draw any conclusion. However, due to a limited number of cases, it is really difficult to find a cutoff value of cytokines to be established as a diagnostic or prognostic marker. The development of an animal model is an alternative to solve this problem and requires a high degree of attention of researchers working in the area. However, despite these limitations, cytokine has certain usefulness as a supportive biomarker to predict the T-lymphoma cell progression and diagnosis. These cytokines may be used as a corroborative marker along with other clinical symptoms and outcomes to estimate the treatment responsiveness and disease progression, but certainly, it warrants more attention from clinicians and researchers.

## 7 Conclusion

As of other cancer cells, the classical T-cell lymphoma TME constitutes various immune-active non-neoplastic cells like lymphocytes, mast cells, macrophages, and eosinophils. These cells may even outnumber the neoplastic cells (31–33). Although the biology of real interaction between the T-lymphoma cells and other cells of the TME is not fully elucidated, the role of cytokine-mediated signaling is getting much attention (35). T-lymphoma cells undergo metabolic alternation to fulfill the ever-increasing demand of ATP, NADH, and NADPH bio-synthesis to support their growth and proliferation. It has been established that in T-cell lymphomas as with other cancers, all the neoplastic cells are not in the same state metabolically (31, 39). The ever-changing TME exerts selective pressure on T-lymphoma cells. Due to the Warburg effect, tumor cells release an increasing amount of lactate in the tumor microenvironment resulting in acidosis and hypoxia (328). Cytokines play an important role in the development of the TME and metabolic reprogramming in T-cell lymphoma (40, 43, 87). The cytokine milieu of the TME dictates the tumor progression, and mutation in cytokine signaling protein significantly favors the tumor development. In addition, TCR- and CD28-mediated signaling and cytokine receptor signaling are very crucial for proper T-cell activation and proliferation. Cytokines like IL-2 and IL-7 are well-established mediators of T-cell lymphoma metabolic reprogramming (38, 60, 277). Alternation in downstream signaling of cytokine receptors has been reported in various types of T-cell lymphomas, which has been discussed in the various sections of the review. The specific mutation in the AKT-P13K and JAK/STAT pathways associated with cytokine receptors has been reported. These alternations ultimately lead to much-needed changes in metabolism to sustain tumor growth and proliferation. Apart from metabolic reprogramming, cytokine-mediated signaling plays an important role in the overall cellular compositions of the TME, as discussed in various sections of this review. T-cell lymphoma development depends upon various extrinsic as well as intrinsic factors, and cytokines have certainly an important role in coordinating these extrinsic and intrinsic factors resulting in T-cell lymphoma development and progression. The cytokines released from different tumor-infiltrating lymphocytes have an important role in the cross-talk between T-lymphomas and their corresponding TME (35). Although T-lymphoma cells resemble metabolically activated T cells, the mutation in the genes related to cytokine signaling has been observed in the development of almost all subtypes of T-cell lymphomas. Thus, the importance of cytokine milieus of the TME in T-lymphoma progression is further needed to be explored for developing a new therapeutic target against T-cell lymphomas. Elucidation of the role of cytokine in tumor development will help in finding T-lymphoma-specific biomarkers for early diagnosis of T-lymphoma and to monitor the disease progression along with in follow-up to the treatment response.

## Author contributions

Corresponding and joint first authors from Guru Ghasidas Vishwavidyalaya wrote and edited the manuscript. All other co-authors contributed intellectually to the designing, editing, and proofreading of the manuscript. All authors contributed to the article and approved the submitted version.
